# Comparison of cone-beam computed tomography with photon-counting detector computed tomography for dental implant surgery

**DOI:** 10.1186/s40729-025-00611-z

**Published:** 2025-03-13

**Authors:** Adib Al-Haj Husain, Victor Mergen, Silvio Valdec, Nadin Al-Haj Husain, Bernd Stadlinger, Harald Essig, Thomas Frauenfelder, Peter Kessler, Suen An Nynke Lie, Hatem Alkadhi, Sebastian Winklhofer

**Affiliations:** 1 Clinic of Cranio-Maxillofacial and Oral Surgery, Center of Dental Medicine, University of Zurich, Plattenstrasse 11, Zurich, 8032 Switzerland; 2 Department of Cranio-Maxillofacial and Oral Surgery, University Hospital Zurich, University of Zurich, Zurich, Switzerland; 3 Department of Neuroradiology, Clinical Neuroscience Center, University Hospital Zurich, University of Zurich, Zurich, Switzerland; 4 Department of Cranio-Maxillofacial Surgery, GROW School for Oncology and Reproduction, Maastricht University Medical Centre, Maastricht, The Netherlands; 5 Diagnostic and Interventional Radiology, University Hospital Zurich, University of Zurich, Zurich, Switzerland; 6 Department of Reconstructive Dentistry and Gerodontology, School of Dental Medicine, University of Bern, Bern, Switzerland; 7 Department of Radiology, Hirslanden Zurich, Zurich, Switzerland

**Keywords:** Dental implant, Computer-guided surgery, Cone-beam computed tomography, Photon-counting detector computed tomography, Low-dose protocol, Radiation protection, Oral surgery, Maxillofacial surgery

## Abstract

**Purpose:**

To compare cone-beam computed tomography (CBCT) with photon-counting detector computed tomography (PCD-CT) at equivalent radiation doses, focusing on qualitative and quantitative parameters relevant to dental implant surgery.

**Methods:**

This ex vivo comparative study of porcine specimens assessed five imaging protocols with both CBCT and PCD-CT at three effective radiation dose levels (high: 360µSv, standard: 145µSv, low: 20µSv) to evaluate image quality, artifact burden, metal artifact susceptibility, and quantitative bone measurements in the mandibular region. Three blinded readers analyzed the data using a 5-point Likert scale (5 = highest to 1 = lowest rating) and performed linear bone measurements at implant planning sites. Statistical analysis included descriptive statistics and inter-reader reliability assessment using intraclass correlation coefficients (ICC).

**Results:**

Each reader evaluated 30 data sets (12 CBCT, 18 PCD-CT), with 24 implant planning sites per imaging protocol. High-dose PCD-CT demonstrated the best image quality and diagnostic interpretability (4.89 ± 0.27), followed by standard-dose PCD-CT and CBCT (4.50 ± 0.73; 4.33 ± 0.61), with low-dose protocols showing intermediate quality with higher artifact burden. In comparison to CBCT, PCD-CT demonstrated superior performance in reducing implant-induced artifacts across all protocols. Quantitative bone measurements showed minimal variability, meeting clinical precision requirements for computer-assisted implant surgery. Both qualitative (ICCs:0.70–0.89; *p* < 0.001) and quantitative (ICCs:0.79–1; *p* < 0.001) analyses demonstrated high reliability, regardless of the reader’s experience.

**Conclusions:**

PCD-CT demonstrated superior image quality and reduced artifacts compared with CBCT at all radiation dose levels. These findings highlight PCD-CT’s potential to enhance implant planning and improve clinical outcomes with reduced radiation exposure while maintaining diagnostic accuracy.

## Background

In the event of tooth loss, several potential treatment options are available to address the resulting gap and its impact on multiple domains of oral health-related quality of life (OHRQoL). Implants have been demonstrated to offer the most favorable long-term outcomes compared to other restorative procedures, with a success rate exceeding 95% after ten years [1]. Achieving a favorable surgical and prosthetic outcome in dental implant surgery requires the implementation of an individualized, multidisciplinary therapeutic strategy that is founded upon the findings of a comprehensive clinical and radiological assessment.

Cone-beam computed tomography (CBCT) is frequently utilized as an adjunct to conventional two-dimensional radiography in surgical treatment planning. The exposure levels range from approximately 18 to 200 µSv per scan, depending on the indication, exposure settings, and region of interest [2]. The integration of CBCT into routine clinical workflows is supported by its accuracy and reliability in cross-sectional structural analysis of bone density and volume [3–6]. Implant planning using CBCT enables precise implant positioning, prosthetically guided placement, and the prevention of damage to critical structures such as neurovascular structures or the maxillary sinus [5, 7]. Additionally, CBCT helps prevent implant failure caused by inadequate bone volume surrounding the implant or improper placement due to poor bone structure [8].

The increasing use of CBCT scans, while providing valuable perioperative insights into surgically relevant parameters at implant sites, raises concerns about cumulative radiation exposure, particularly in genetically susceptible younger patients. Repeated imaging may elevate the risk of adverse health effects, potentially increasing the likelihood of developing thyroid cancer and meningiomas [9, 10]. In light of the proposed shift from the “as low as reasonably achievable” (ALARA) principle to the “as low as diagnostically acceptable” (ALADA) principle [11] and its subsequent adaptation to “as low as diagnostically acceptable being indication-oriented and patient specific” (ALADAIP) principle [12], studies are currently examining low-dose CBCT protocols in the context of dental implant surgery [13]. Preliminary findings suggest the feasibility of using low-dose CBCTs perioperatively compared to standard-dose CBCTs [14]. However, there are limitations in the generalizability of these results, particularly regarding reliability and validity in the posterior mandible and the standardization of measurement sites [6, 15].

Photon-counting detectors (PCD) are the most recent development in computed tomography (CT) and employ semiconductors to directly convert incoming X-ray photons into electrical signals, providing superior spatial resolution, less electronic noise, enhanced contrast-to-noise ratio, and distinctive spectral features [16]. PCD-CT addresses shortcomings of CBCT in dental implant imaging, such as low spatial resolution, deficiencies in soft-tissue contrast, and increased susceptibility to metal artifacts [17]. In the ultra-high-resolution mode of PCD-CT, detector pixels measure 0.151 × 0.176 mm^2^ at the isocenter, which translates to a maximum spatial in-plane image resolution of 0.11 mm and a maximum through-plane resolution of 0.16 mm [18, 19]. Thus, PCD-CT scans offer spatial resolutions of < 200 μm, comparable to or even superior to dental CBCT, and provide high-quality volumetric imaging with enhanced hard and soft tissue contrast and shorter scan times [20]. The use of PCD-CT in dentomaxillofacial imaging has been investigated in only a few studies, yet the findings are promising, including accurate anatomical depiction, improved reduction of metal artifacts from dental implants [21], and improved radiation dose efficiency, with doses as low as a quarter of those used in CBCT [22]. A feasibility study directly compared artifacts from a titanium implant using CBCT and PCD-CT, supporting PCD-CT’s superior artifact-reduction potential [23]. To the best of the author’s knowledge, this is the first study to directly compare PCD-CT with CBCT in the same samples at equivalent radiation doses for dental implant imaging.

The objective of this ex vivo study was to compare CBCT with PCD-CT at equivalent radiation doses, focusing on qualitative and quantitative parameters relevant to dental implant surgery. Given PCD-CT’s potential for superior artifact reduction, improved radiation dose efficiency, and high image quality at lower doses, this study investigates its ability to improve diagnostic accuracy, minimize implant planning errors, and thus support more precise clinical decision making.

## Methods

### Study design and ethics

In this ex vivo comparative study, six pig mandibles were obtained from a local butcher’s shop in Zurich, Switzerland. Twelve dental implants were placed in the pig mandibles in a randomized order. Each mandible received two implants, with one implant placed in each quadrant between the canine and the first premolar. The procedure was conducted by an experienced senior physician (S.V., a board-certified oral surgeon with 11 years of experience). The study utilized implants from four commonly used brands in clinical practice: Dentsply Sirona (*Astra Tech OsseoSpeed*^®^*EV 4.2 S, Mölndal, Sweden*), Nobel Biocare (*NobelActive*^®^*TiUltra*™, *Göteborg, Sweden*), Straumann (*Standard Plus SLActive*^®^, *Basel, Switzerland*), and Thommen Medical (*SPI*^®^*ELEMENT Implantat RC INICELL*^®^, *Grenchen, Switzerland*). Due to ethical considerations and radiation safety reasons, conducting this study in a living organism was not feasible [13]. Nevertheless, pig cadavers, which closely align with the human oral and maxillofacial system, are widely regarded as suitable animal models for orofacial research. Consequently, they were used as an alternative in this study [24]. A formal declaration of non-responsibility was provided by the Office of Animal Welfare and 3R of the University of Zurich, confirming that all experiments comply with the Swiss federal guidelines for the use of animals in experimental research. This article’s reporting complies with the ARRIVE (Animal Research: Reporting of In Vivo Experiments) guidelines.

### Image acquisition

#### CBCT data acquisition

All mandibles were imaged using the Orthophos SL three-dimensional (3D) scanner (Dentsply-Sirona, Bensheim, Germany) following the manufacturer’s predefined standard and low-dose CBCT protocols. The mandibles were centrally positioned and aligned on a platform using the scanner’s positioning lights. To replicate in vivo conditions as accurately as possible, a cold pack (12 × 29 cm, GELLO Geltechnik GmbH, Ahaus, Germany) was placed at the center of each mandible to simulate the presence of soft tissue. The standard-dose imaging protocol was conducted with 85 kV, 13 mA, 4.4 s exposure time, 160 μm pixel size, and an 11 × 10 cm field of view, with an effective dose of 145 µSv. The low-dose imaging protocol used 85 kV, 13 mA, 2.2 s exposure time, 160 μm pixel size, and an 11 × 10 cm field of view, with an effective dose of 20 µSv.

#### PCD-CT data acquisition

Scans were acquired on a first-generation dual-source PCD-CT system (NAEOTOM Alpha; Siemens Healthineers AG, Forchheim, Germany) equipped with two cadmium telluride detectors using the ultra-high resolution mode with a detector collimation of 120 × 0.2 mm. The tube voltage was set to 140 kV, using tin pre-filtration, and the pitch factor was 0.85. Radiation doses of the PCD-CT scans were varied to match the doses of the CBCT scans, i.e., equivalent to the standard-dose protocol by adjusting the volume CT dose index (CTDI_vol_) to 2.4 mGy, resulting in a dose-length-product (DLP) of 61 mGy $\:\bullet\:$ cm and an effective dose of 122 µSv (using a conversion factor of 0.002 mSv $\:\bullet\:\:$mGy^− 1^$\:\bullet\:$ cm^− 1^ according to [25]), and equivalent to the low-dose protocol the by adjusting the CTDI_vol_ to 0.4 mGy, resulting in DLP of 10 mGy $\:\bullet\:$ cm and an effective dose of 20 µSv. In addition, CTDI_vol_ was adjusted to 7.0 mGy, resulting in a DLP of 180 mGy $\:\bullet\:$ cm and an effective dose of 360 µSv.

Scans were reconstructed as polychromatic images (T3D) with a slice thickness of 0.2 mm and an increment of 0.2 mm. The sharp kernel Hr76 was applied, and the matrix size was 1024 × 1024 pixels.

### Image analysis

CBCT and PCD-CT image data were evaluated on a 2-megapixel high-quality liquid-crystal display. A total of 30 scans, compromising 12 CBCT and 18 PCD-CT scans, were assessed by three readers with different levels of experience and specialization. Reader A (A.A.H.) is a resident in oral surgery with four years of experience; Reader B (N.A.H.) is an attending physician, board-certified in reconstructive dentistry, holding a Master of Advanced Studies in reconstructive and implant dentistry, with nine years of experience; and Reader C (S.V.) is a senior physician, board-certified in oral surgery, with 11 years of experience. Prior to the evaluation, a calibration session was conducted to standardize the assessment process. Each examiner received instructions from one of the principal investigators (A.A.H.). To address and eliminate any potential ambiguities, three randomly selected protocols were evaluated. To ensure objective and unbiased assessments, all readers performed the evaluations of the various scans, including protocol and modality, in randomized order. They were blinded to each other’s evaluations, the imaging protocols (low-dose, standard-dose, and high-dose), and the types of implants used.

### Qualitative measurements

The objective analysis of the overall technical image quality and the burden of technical artifacts was graded using a modified 5-point Likert scale, as previously described by Dillinger et al. in the context of PCD-CT dental implant research [26]: 5, excellent image quality with full diagnostic capabilities; 4, good image quality with sufficient diagnostic capabilities; 3, intermediate image quality with restricted diagnostic capabilities; 2, poor image quality with very limited diagnostic capabilities; and 1, indicated very poor image quality, allowing no diagnostic use. For technical artifact burden, the following scale was employed: 5, no artifacts; 4, minimal streaks; 3, intermediate streaks; 2, pronounced streaks; and 1, massive artifacts.

The prevalence of hyperdense and hypodense artifacts caused by the placed dental implants was assessed using the following modified 5-Likert Scale according to Patzer et al. [27]: 5, absent/almost absent, no metallic artifact; 4, minor, metallic artifacts are minimal and do not affect scan quality or diagnosis; 3, moderate, metallic artifacts are present and affect overall scan quality, but they do not interfere with the evaluation of adjacent anatomy or diagnosis; 2, pronounced, metallic artifacts are present and affect overall scan quality, and interfere with the evaluation of adjacent anatomy and diagnosis; 1, severe, metallic artifacts are significant, severely affect scan quality, obscure adjacent anatomy, and compromise diagnosis.

Additionally, the diagnostic interpretability of soft and hard tissue was evaluated using the following Likert scale [27]: 5, fully diagnostic; 4, minor artifacts with marginal impairment of diagnostic interpretability; 3, artifacts with impaired, mediocre diagnostic interpretability; 2, artifacts with significantly impaired diagnostic interpretability; 1, insufficient interpretability due to artifacts.

### Quantitative measurements

Quantitative analysis of the CBCT and PCD-CT scans relevant to dental implant treatment planning in the posterior mandible was performed on a dedicated, commercially available software (OnDemand 3D, Cybermed, Seoul, Korea). Linear bone measurements in dental implant surgery were conducted according to the method proposed by Kaaber et al. [28]. The image generation process was initiated using the standard-dose CBCT images, where the most appropriate reconstructions, both in the sagittal and coronal planes, were selected for the visualization of the implant site. Subsequently, the defined implant site was replicated using the low-dose CBCT and high, standard, and low PCD-CT images. To obtain measurements at the same site in the specified object, the investigators were instructed to take the measurement along the vertical and horizontal guiding lines. The software automatically sets the window width and window plane for the standard-dose CBCT protocol, thereby standardizing the viewing conditions of the image for all the protocols in each modality. The assessment included measuring bone height, defined as the vertical distance from the alveolar crest to the upper border of the mandibular canal, along the vertical guiding line, and bone width, defined as the horizontal distance three millimeters apical to the alveolar crest between the buccal and lingual cortical boundaries, measured along the horizontal guiding line [23]. The measurements, which were taken in millimeters, were conducted by the same three readers. Accordingly, two implant sites were selected from each quadrant of the posterior mandible, resulting in four bone height and width measurements per mandible for each imaging protocol and reader. If the investigator was uncertain about performing the measurements due to the suboptimal image quality or the presence of artifacts, the case could be classified as unsuitable for measurement. To prevent recall bias, each measurement was repeated after a three-week interval. In the event of a discrepancy between the initial and subsequent measurements, the mean of the two values was calculated.

### Statistical analysis

Descriptive statistics were used to analyze the qualitative data obtained, including overall technical image quality, the burden of technical artifacts, artifacts caused by the placed dental implants, and diagnostic interpretability of soft and hard tissue. The analysis involved calculations of means, standard deviations (SD), medians, minimums, maximums, and ranges.

Additionally, inter-reader agreement for the assessed qualitative variables was determined and expressed as a percentage agreement or by analyzing the intraclass correlation coefficient (ICC) type 2:1 and the 95% confidence interval (CI) based on the agreement according to the 2-way random model. In accordance with the selected 95% CI, the ICC values and their agreement beyond chance were interpreted as follows: poor (< 0.5), moderate (0.5–0.75), good (0.75–0.9), and excellent (> 0.9) [29]. Regarding quantitative parameters, the mean difference among readers and the inter-reader reliability of absolute length measurements were evaluated and expressed as ICCs. All statistical analyses were conducted with a significance level of α = 0.05. All statistical analyses were performed using IBM SPSS Statistics software (version 29.0, IBM Chicago, IL, USA).

## Results

Three readers qualitatively evaluated 30 DICOM data sets (12 CBCT, 18 PCD-CT), while each reader quantitatively assessed 24 implant planning sites in the posterior mandible per imaging protocol, resulting in a total of 120 implant sites per reader.

### Qualitative results

The mean overall technical image quality ranged from good to excellent, demonstrating full diagnostic capabilities for high-dose PCD-CT (4.89 ± 0.27), standard-dose PCD-CT (4.50 ± 0.73), and standard-dose CBCT (4.33 ± 0.61). In contrast, low-dose protocols for both modalities showed intermediate image quality with restricted diagnostic capabilities (CBCT: 3.22 ± 0.35; PCD-CT: 3.17 ± 0.63).

The artifact burden was most pronounced among all readers in the low-dose protocols for both imaging modalities. Notably, standard-dose PCD-CT (4.11 ± 0.27) exhibited lower artifact susceptibility compared to standard-dose CBCT (3.67 ± 0.52), with the high-dose PCD-CT protocol demonstrating the least artifact burden (4.89 ± 0.27).

**Fig. 1:**
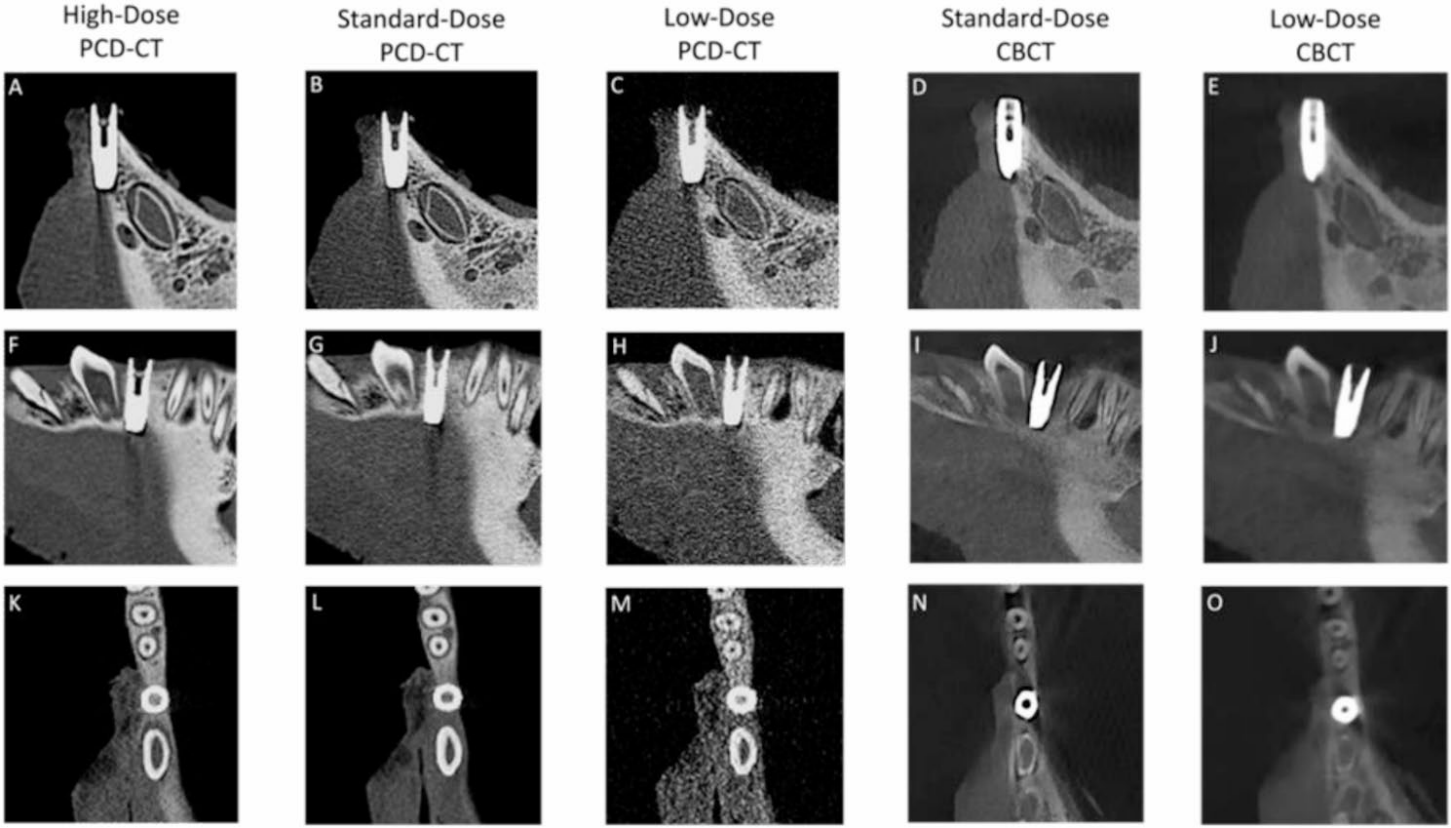
This figure illustrates the performance of photon-counting computed tomography (PCD-CT) and cone-beam computed tomography (CBCT) at equivalent radiation exposure levels. The qualitative parameters assessed include technical image quality, susceptibility to technical artifacts, and implant-induced artifacts. In this pig mandible, the implant is placed in each quadrant between the canine and the first premolar, positioned at the bone-soft tissue interface to assess artifact susceptibility and occurrence on both the osseous and soft tissue sides. **A**–**E** show coronal reconstructions, **F**–**J** sagittal reconstructions, and **K**–**O** axial reconstructions for each imaging modality. High-dose and standard-dose PCD-CT demonstrated excellent image quality with a marked reduction in implant-induced artifacts. Standard-dose and low-dose PCD-CT outperformed CBCT in both hard and soft tissue visualization

**Fig. 2:**
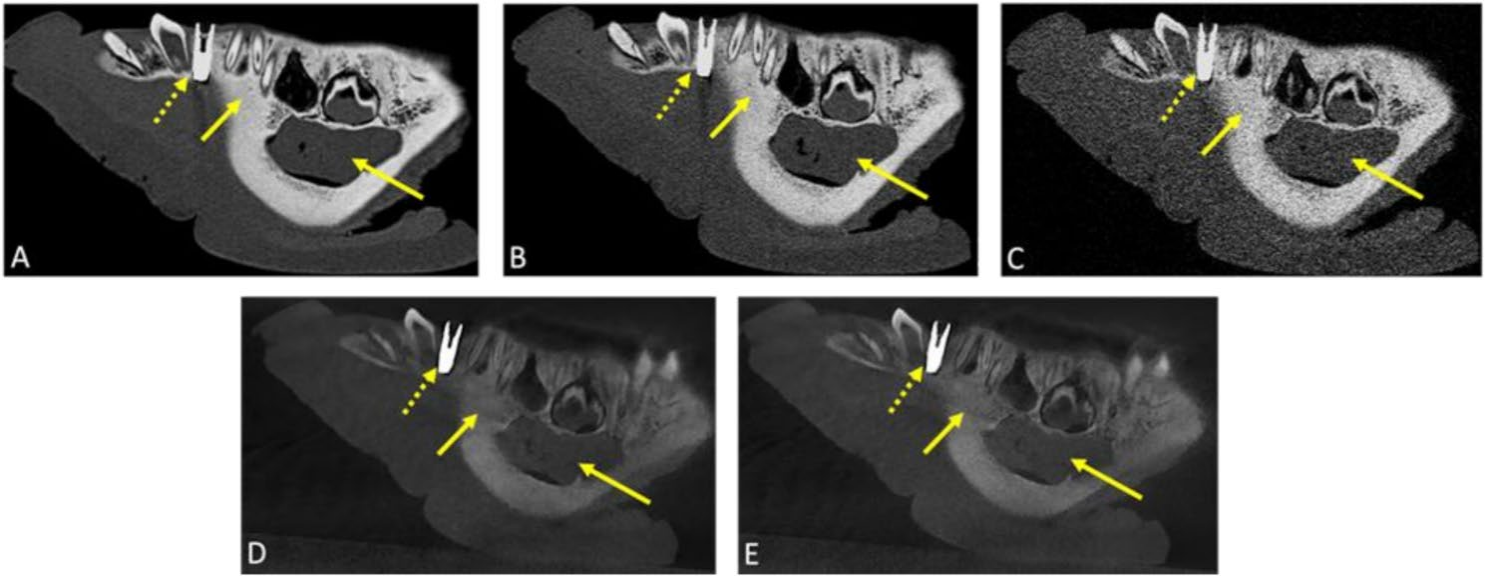
The figure depicts sagittal reconstructions of photon-counting computed tomography (PCD-CT) scans (**A**: high-dose; **B**: standard-dose; **C**: low-dose) and cone-beam computed tomography (CBCT) scans ((**D**: standard-dose; **E**: low-dose) of the same pig mandible. The standard-dose and low-dose PCD-CT scans were acquired at radiation exposure levels equivalent to those of the corresponding CBCT scans, allowing for direct comparison. The dotted arrow indicates the placed implant in each quadrant between the canine and the first premolar, while the short arrow highlights the visualization of osseous structures. The longer arrow indicates the location of the neurovascular bundle within the mandibular canal, within the mandibular canal, representing the visualization of soft tissues representing the visualization of soft tissues

Hypodense and hyperdense artifacts related to the material properties of implants were either absent or minimal in high-dose PCD-CT scans (4.67 ± 0.52). Standard-dose PCD-CT scans demonstrated minor metallic artifacts (4.17 ± 0.82), not compromising image quality or diagnostic accuracy. In contrast, standard-dose CBCT exhibited moderate metallic artifacts (3.45 ± 0.59) that significantly reduced overall scan quality (Figs. 1 and 2).

**Fig. 3:**
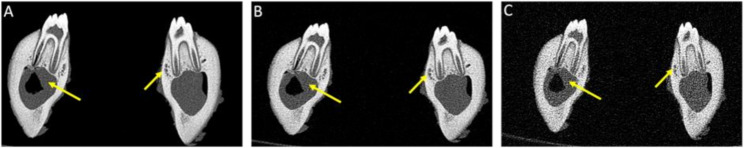
Coronal reconstructions of photon-counting computed tomography (PCD-CT) scans are shown, including (**A**) a scan acquired at a high-dose levels, (**B**) a scan acquired at standard-dose levels, and (**C**) a scan acquired at low-dose levels. The low-dose scans use radiation doses equivalent to those in conventional cone-beam computed tomography (CBCT), enabling direct comparisons for dental implant planning. The long arrow highlights osseous structures, demonstrating a cross-sectional analysis of bone density and volume, which are key parameters for implant placement. Additionally, the scans effectively visualize critical soft tissue structures, such as the mandibular canal (indicated by the short arrow), offering substantial advantages for accurate implant placement in prosthetically guided procedures. This helps minimize the risk of damaging vital structures, including neurovascular bundles and the maxillary sinus

The diagnostic interpretability of both hard and soft tissues was generally rated as fully diagnostic, with minor artifacts causing only marginal impairment. Among the evaluated protocols, the high-dose PCD-CT achieved the highest scores for diagnostic interpretability (hard tissue: 4.83 ± 0.41; soft tissue: 4.44 ± 0.52). Standard-dose PCD-CT also outperformed the radiation dose-equivalent CBCT protocol for both hard tissue (4.56 ± 0.53 vs. 3.33 ± 0.52) and soft tissue (3.78 ± 0.76 vs. 2.44 ± 0.54) visualization (Fig. 3). Results of all assessed qualitative parameters are presented in Table 1.

**Table 1:** This table summarizes the mean scores from qualitative assessments conducted by three independent readers, each with distinct subspecialties and experience levels: reader A (resident with 4 years of experience), reader B (board-certified attending with 9 years of experience), and reader C (board-certified attending with 11 years of experience). The evaluation parameters included technical image quality, susceptibility to artifacts, implant-induced artifacts, and the visualization of hard and soft tissues. Scores were assigned using a modified 5-point likert scale, with 5 indicating the optimal result and 1 indicating the least favorable result within each category

	Imaging Protocol	Reader A	Reader B	Reader C	Average
Technical Image Quality (Mean ± SD (Median))	SD-CBCT LD-CBCT	4.33 ± 0.52 (4) 3.33 ± 0.52 (4)	4.5 ± 0.55 (4.5) 3 ± 0 (3)	4.17 ± 0.75 (4) 3.33 ± 0.52 (4)	4.33 ± 0.61 3.22 ± 0.35
	HD-PCD-CT SD-PCD-CT LD-PCD-CT	5 ± 0 (5) 4.5 ± 0.84 (5) 3.17 ± 0.75 (3)	4.83 ± 0.41 (5) 4.67 ± 0.52 (5) 3 ± 0.63 (3)	4.83 ± 0.41 (5) 4.33 ± 0.82 (4.5) 3.33 ± 0.52 (3)	4.89 ± 0.27 4.5 ± 0.73 3.17 ± 0.63
Technical Artifacts (Mean ± SD (Median))	SD-CBCT LD-CBCT	3.67 ± 0.52 (4) 3.17 ± 0.41 (3)	3.67 ± 0.52 (4) 3 ± 0 (3)	3.67 ± 0.52 (4) 3.33 ± 0.52 (3)	3.67 ± 0.52 3.17 ± 0.31
	HD-PCD-CT SD-PCD-CT LD-PCD-CT	4.83 ± 0.41 (5) 4 ± 0 (4) 3.5 ± 0.55 (3.5)	5 ± 0 (5) 4.17 ± 0.41 (4) 3.67 ± 0.82 (3.5)	4.83 ± 0.41 (5) 4.17 ± 0.41 (4) 3.67 ± 0.52 (4)	4.89 ± 0.27 4.11 ± 0.27 3.61 ± 0.63
Artifacts associated with dental implants (Mean ± SD (Median))	SD-CBCT LD-CBCT	3.5 ± 0.55 (3.5) 2.33 ± 0.52 (2)	3.67 ± 0.82 (3.5) 2.17 ± 0.75 (2)	3.17 ± 0.41 (3) 2.33 ± 0.52 (2)	3.45 ± 0.59 2.28 ± 0.6
	HD-PCD-CT SD-PCD-CT LD-PCD-CT	4.67 ± 0.52 (5) 4.33 ± 0.82 (4.5) 3.3 ± 0.82 (3.5)	4.67 ± 0.52 (5) 4.17 ± 0.75 (4) 3.17 ± 0.76 (3)	4.67 ± 0.52 (5) 4 ± 0.89 (4) 2.83 ± 0.52 (2)	4.67 ± 0.52 4.17 ± 0.82 3.1 ± 0.7
Hard Tissue Imaging (Mean ± SD (Median))	SD-CBCT LD-CBCT	3.33 ± 0.52 (3) 2.87 ± 0.81 (2.5)	3.33 ± 0.52 (3) 3 ± 0.63 (3)	3.33 ± 0.52 (3) 3 ± 0.63 (3)	3.33 ± 0.52 2.95 ± 0.69
	HD-PCD-CT SD-PCD-CT LD-PCD-CT	4.83 ± 0.41 (5) 4.67 ± 0.52 (5) 3.33 ± 0.52 (3)	4.83 ± 0.41 (5) 4.5 ± 0.55 (4.5) 4.17 ± 0.41 (3)	4.83 ± 0.41 (5) 4.5 ± 0.55 (4.5) 3.17 ± 0.41 (3)	4.83 ± 0.41 4.56 ± 0.54 3.56 ± 0.45
Soft Tissue Imaging (Mean ± SD (Median))	SD-CBCT LD-CBCT	2.5 ± 0.55 (2.5) 2.33 ± 0.52 (2)	2.33 ± 0.52 (2) 2.16 ± 0.41 (2)	2.5 ± 0.55 (2.5) 2.33 ± 0.52 (2)	2.44 ± 0.54 2.27 ± 0.48
	HD-PCD-CT SD-PCD-CT LD-PCD-CT	4.33 ± 0.52 (4) 3.67 ± 0.82 (3.5) 2.83 ± 0.41 (3)	4.33 ± 0.52 (4) 3.67 ± 0.82 (3.5) 2.83 ± 0.41 (3)	4.67 ± 0.52 (5) 4 ± 0.63 (4) 2.83 ± 0.41 (3)	4.44 ± 0.52 3.78 ± 0.76 2.83 ± 0.41

CBCT: Cone-Beam Computed Tomography; PCD-CT: Photon-Counting Computed Tomography; HD: High-Dose; SD: Standard Dose; LD: Low Dose

The inter-reader agreement for all parameters ranged from moderate to good across all imaging modalities and protocols, with intraclass correlation coefficient (ICC) values reaching up to 0.89 (range: 0.70–0.89). The highest reliability was observed in the high-dose PCD-CT and the standard-dose PCD-CT and CBCT protocols, whereas the low-dose protocols yielded the lowest ICC values. All parameters demonstrated statistically significant agreement, with *p*-values < 0.05. Detailed inter-reader agreement for each parameter, assessed across the three readers within each modality and protocol, is presented in Table 2.

**Table 2:** The inter-reausing the intraclass correlation coefficient (ICC) with a two-sided 95% confidence interval (CI)der agreement among the three readers - reader A (resident), reader B (attending), and reader C (attending) - was evaluated for each qualitative parameter and expressed as percentage agreement. Additionally, the overall agreement across all parameters for the specific imaging modality and protocol was quantified

Inter-Reader percentage agreement	Imaging Protocol	Reader A and B	Reader B and C	Reader C and A	Average
Technical Image Quality	SD-CBCT	83.3%	66.7%	83.3%	77.8%
	LD-CBCT	66.7%	66.7%	83.3%	72.2%
	HD-PCD-CT	83.3%	100%	83.3%	88.9%
	SD-PCD-CT	83.3%	83.3%	66.7%	77.8%
	LD-PCD-CT	83.3%	66.7%	83.3%	77.8%
Technical Artifacts	SD-CBCT	100%	83.3%	100%	94.4%
	LD-CBCT	83.3%	83.3%	100%	88.9%
	HD-PCD-CT	100%	100%	83.3%	94.4%
	SD-PCD-CT	83.3%	100%	83.3%	88.9%
	LD-PCD-CT	83.3%	83.3%	83.3%	83.3%
Artifacts associated with dental implants	SD-CBCT	83.3%	100%	66.7%	83.3%
	LD-CBCT	66.7%	83.3%	100%	83.3%
	HD-PCD-CT	100%	100%	100%	100%
	SD-PCD-CT	83.3%	83.3%	83.3%	83.3%
	LD-PCD-CT	100%	100%	83.3%	94.4%
Hard Tissue Imaging	SD-CBCT	100%	100%	100%	100%
	LD-CBCT	83.3%	83.3%	83.3%	83.3%
	HD-PCD-CT	100%	100%	100%	100%
	SD-PCD-CT	83.3%	100%	83.3%	88.9%
	LD-PCD-CT	100%	100%	83.3%	94.4%
Soft Tissue Imaging	SD-CBCT	50%	83.3%	83.3%	72.2%
	LD-CBCT	83.3%	83.3%	66.7%	77.8%
	HD-PCD-CT	83.3%	66.7%	83.3%	77.8%
	SD-PCD-CT	66.7%	66.7%	66.7%	66.7%
	LD-PCD-CT	50%	66.7%	66.7%	61.1%
Inter-Reader agreement (ICC (95% CI))	**Imaging Protocol**	**Reader A and B**	**Reader B and C**	**Reader C and A**	**Average**
All Parameters	SD-CBCT	0.88 (0.77–0.94); *p* < 0.001	0.92 (0.85–0.96); *p* < 0.001	0.89 (0.79–0.95); *p* < 0.001	0.89
	LD-CBCT	0.67 (0.49–0.855); *p* < 0.001	0.71 (0.61–0.89); *p* < 0.001	0.72 (0.45–0.91); *p* < 0.001	0.7
	HD-PCD-CT	0.84 (0.69–0.92); *p* < 0.001	0.85 (0.69–0.93); *p* < 0.001	0.91 (0.82–0.95); *p* < 0.001	0.87
	SD-PCD-CT	0.79 (0.58–0.9); *p* < 0.001	0.84 (0.67–0.92); *p* < 0.001	0.82 (0.62–0.91); *p* < 0.001	0.82
	LD-PCD-CT	0.69 (0.46–0.89); *p* < 0.001	0.83 (0.68–0.92); *p* < 0.001	0.73 (0.43–0.82); *p* < 0.001	0.75

CBCT: Cone-Beam Computed Tomography; PCD-CT: Photon-Counting Computed Tomography; HD: High-Dose; SD: Standard-Dose; LD: Low-Dose

Regarding manufacturer-specific differences in implant susceptibility to artifacts, high-dose PCD-CT consistently produced the fewest artifacts and the highest image quality across all implant manufacturers. Notably, no specific implant subtype was associated with a significantly higher incidence of artifacts (Table 3).

**Table 3:** The following table presents the mean scores for all three readers (Reader A (resident), reader B (attending), and reader C (attending)) on the image quality and prevalence of hyperdense and hypodense artifacts caused by the implants utilized in this study, which were from four commonly used brands (Dentsply Sirona, nobel biocare, Straumann, and Thommen Medical) in clinical practice. The scores are based on the modified 5-point likert scale, which ranges from 5 (indicating excellent image quality or the absence or near absence of artifacts) to 1 (indicating poor image quality or the presence of severe artifacts)

All Readers (A, B, and C)	Imaging Protocol	Image Quality (Mean ± SD)	Artifacts caused by implants (Mean ± SD, (Median))	Artifacts caused by implants - Minimum	Artifacts caused by implants - Maximum
Dentsply Sirona (Astra Tech OsseoSpeed® EV 4.2 S, Mölndal, Sweden)	SD-CBCT	4.58 ± 0.51 (5)	3.33 ± 0.49 (3)	3	4
	LD-CBCT	3.17 ± 0.39 (3)	2.5 ± 0.52 (2.5)	2	3
	HD-PCD-CT	5 ± 0 (5)	5 ± 0 (5)	5	5
	SD-PCD-CT	4.67 ± 0.65 (5)	4.41 ± 0.51 (4)	3	5
	LD-PCD-CT	3.17 ± 0.39 (3)	3.75 ± 0.45 (4)	3	4
Nobel Biocare (NobelActive® TiUltra™, Göteborg, Sweden)	SD-CBCT	3.83 ± 0.41 (4)	3.17 ± 0.41 (3)	3	4
	LD-CBCT	3.17 ± 0.41 (3)	2.13 ± 0.41 (2)	1	3
	HD-PCD-CT	4.83 ± 0.41 (5)	4.33 ± 0.52 (4)	4	5
	SD-PCD-CT	4.67 ± 0.52 (5)	3.83 ± 0.52 (3)	3	4
	LD-PCD-CT	3.17 ± 0.98 (3.5)	2.93 ± 0.75 (3)	2	4
Straumann (Standard Plus SLActive®, Basel, Switzerland)	SD-CBCT	3.9 ± 0.45 (4)	3.25 ± 0.42 (3)	3	4
	LD-CBCT	3.1 ± 0.38 (3)	2.2 ± 0.43 (2)	1	3
	HD-PCD-CT	4.83 ± 0.41 (5)	4.6 ± 0.4 (4)	4	5
	SD-PCD-CT	4.67 ± 0.52 (5)	3.9 ± 0.5 (3.5)	3	4
	LD-PCD-CT	3.17 ± 0.98 (3.5)	3.15 ± 0.7 (3)	1	4
Thommen Medical (SPI®ELEMENT Implantat RC INICELL®, Grenchen, Switzerland)	SD-CBCT	4.27 ± 0.52 (5)	3.17 ± 0.41 (3)	3	4
	LD-CBCT	3.3 ± 0.52 (3)	2.5 ± 0.55 (2.5)	2	3
	HD-PCD-CT	5 ± 0 (5)	5 ± 0 (5)	5	5
	SD-PCD-CT	4.17 ± 0.41 (4)	4.17 ± 0.41 (4)	4	5
	LD-PCD-CT	3 ± 0 (3)	3.5 ± 0.55 (3.5)	3	4

CBCT: Cone-Beam Computed Tomography; PCD-CT: Photon-Counting Computed Tomography; HR: High-Dose; SD: Standard-Dose; LD: Low-Dose

### Quantitative results

The quantitative analysis of linear bone measurements in the posterior mandible revealed that one case exhibited non-measurable height measurements, while all cases yielded successfully obtained width measurements. The mean differences among the three readers were minimal for both height and width measurements, demonstrating that both imaging modalities fulfill the clinical precision requirements for implant planning across different radiation levels, regardless of the reader’s experience or subspecialty.

The inter-reader agreement for absolute length measurements was rated as good to excellent across all imaging protocols for both height and width measurements, with ICC ranges of 0.793 to 1.0 and 0.817 to 0.991 (all *p* < 0.001), respectively. A protocol-specific analysis revealed that both high-dose and standard-dose protocols demonstrated higher inter-reader agreement than low-dose protocols, regardless of the imaging modality. Notably, PCD-CT low-dose protocols demonstrated superior performance to CBCT protocols at equivalent radiation doses in most inter-reader comparisons, with the exception of one instance (Tables 4 and 5).

**Table 4:** The mean difference (in millimeters (mm)) and the interobserver reproducibility for absolute length measurements were expressed by the intraclass correlation coefficient (ICC) with a two-sided 95% confidence interval (CI) for bone height measurements, defined as the vertical distance from the alveolar crest to the upper border of the mandibular Canal along the vertical guiding line. A total of 24 measurements were evaluated in each imaging protocol, resulting in a total of 120 implant sites per reader, with three different readers (Reader A, a resident physician; reader B, an attending physician; and reader C, an attending physician) participating in the assessment

Observer pairs	Imaging Protocol	Mean (mm)	SD	Min. (mm)	Max. (mm)	Inter-Reader Agreement - ICC (95% CI)
Reader A – Reader B	SD-CBCT	0.058	0.21	− 0.045	1	1 (1–1); *p* < 0.001
	LD-CBCT	0.001	0.07	-0.117	0.219	0.793 (0.595–0.931); *p* < 0.001
	HD-PCD-CT	0.006	0.005	− 0.008	0.23	0.907 (0.785–0.96); *p* < 0.001
	SD-PCD-CT	− 0.013	0.07	− 0.24	0.15	1 (1–1); *p* < 0.001
	LD-PCD-CT	− 0.005	0.066	− 0.134	0.153	0.825 (0.630–0.923); *p* < 0.001
Reader A – Reader C	SD-CBCT	0.017	0.057	-0.073	0.213	0.892 (0.809–0.967); *p* < 0.001
	LD-CBCT	− 0.008	0.094	− 0.28	0.219	0.833 (0.595–0.931); *p* < 0.001
	HD-PCD-CT	0.002	0.01	− 0.01	0.04	0.998 (0.995–0.999); *p* < 0.001
	SD-PCD-CT	− 0.021	0.071	-0.28	0.03	0.956 (0.901–0.981); *p* < 0.001
	LD-PCD-CT	− 0.02	0.081	− 0.223	0.174	0.904 (0.773–0.96); *p* < 0.001
Reader B – Reader C	SD-CBCT	0.001	0.03	-0.062	0.08	0.966 (0.92–0.986); *p* < 0.001
	LD-CBCT	− 0.009	0.064	-0.27	0.084	0.885 (0.746–0.95); *p* < 0.001
	HD-PCD-CT	0.003	0.01	− 0.01	0.04	0.903 (0.777–0.958); *p* < 0.001
	SD-PCD-CT	− 0.001	0.035	− 0.125	0.047	0.978 (0.948–0.99); *p* < 0.001
	LD-PCD-CT	− 0.014	0.088	− 0.338	0.2	0.902 (0.767–0.959); *p* < 0.001

CBCT: Cone-Beam Computed Tomography; PCD-CT: Photon-Counting Computed Tomography; HD: High-Dose; SD: Standard-Dose; LD: Low-Dose

**Table 5:** The mean difference (in millimeters (mm)) and interobserver reproducibility for absolute length measurements were measured by the intraclass correlation coefficient (ICC) with a two-sided 95% confidence interval (CI) for bone width measurements, defined as the horizontal distance three mm apical to the alveolar crest between the buccal and lingual cortical boundaries measured along the horizontal guiding line. A total of 24 measurements were evaluated in each imaging protocol, resulting in a total of 120 implant sites per reader with three different readers (Reader A, a resident physician; Reader B, an attending physician; and Reader C, an attending physician) participating in the assessment

Observer pairs	Imaging Protocol	Mean (mm)	SD	Min. (mm)	Max. (mm)	Inter-Reader Agreement - ICC (95% CI)
Reader A – Reader B	SD-CBCT	− 0.02	0.06	− 0.26	0.035	0.886 (0.645–0.934); *p* < 0.001
	LD-CBCT	− 0.02	0.074	− 0.22	0.135	0.821 (0.586–0.922); *p* < 0.001
	HD-PCD-CT	− 0.005	0.013	− 0.045	0.005	0.991 (0.979–0.996); *p* < 0.001
	SD-PCD-CT	− 0.006	0.062	− 0.258	0.098	0.897 (0.531–0.912); *p* < 0.001
	LD-PCD-CT	− 0.007	0.036	− 0.109	0.07	0.893 (0.769–0.952); *p* < 0.001
Reader A – Reader C	SD-CBCT	− 0.02	0.074	− 0.22	0.135	0.862 (0.612–0.927); *p* < 0.001
	LD-CBCT	− 0.012	0.07	− 0.150	0.135	0.829 (0.604–0.926); *p* < 0.001
	HD-PCD-CT	− 0.009	0.019	− 0.073	0.005	0.978 (0.95–0.991); *p* < 0.001
	SD-PCD-CT	− 0.021	0.071	− 0.28	0.028	0.871 (0.64–0.964); *p* < 0.001
	LD-PCD-CT	− 0.02	− 0.223	− 0.223	0.174	0.855 (0.693–0.934); *p* < 0.001
Reader B – Reader C	SD-CBCT	− 0.006	0.064	− 0.18	0.123	0.886 (0.645–0.934); *p* < 0.001
	LD-CBCT	0.007	0.035	− 0.064	0.106	0.824 (0.733–0.966); *p* < 0.001
	HD-PCD-CT	0.005	0.017	− 0.071	0.009	0.985 (0.965–0.993); *p* < 0.001
	SD-PCD-CT	− 0.017	0.047	− 0.180	0.02	0.848 (0.681–0.931); *p* < 0.001
	LD-PCD-CT	− 0.063	0.044	− 0.132	0.08	0.817 (0.622–0.916); *p* < 0.001

CBCT: Cone-Beam Computed Tomography; PCD-CT: Photon-Counting Computed Tomography; HD: High-Dose; SD: Standard-Dose; LD: Low-Dose

## Discussion

The objective of this ex vivo study was to evaluate the diagnostic interpretability of PCD-CT imaging protocols compared to clinically established CBCT protocols across varying equivalent radiation exposure levels, focusing on surgically relevant qualitative and quantitative parameters in dental implant surgery. Evaluating 30 reconstructions, high-dose PCD-CT demonstrated the best overall performance, offering superior image quality, minimal artifacts, and excellent diagnostic accuracy. At equivalent radiation exposure levels, PCD-CT performed superior to CBCT, while low-dose protocols of both modalities exhibited intermediate image quality and limited diagnostic capabilities. Both modalities achieved high inter-reader agreement and met clinical quantitative precision requirements regarding implant planning capabilities. However, PCD-CT consistently exhibited superior performance in reducing implant-induced metallic artifacts across all dose levels and implant manufacturers. These findings are in line with previous studies that highlight the advanced capabilities of PCD-CT for dental implant imaging [20, 21].

Dentomaxillofacial imaging constitutes the most frequently performed X-ray-based procedures globally, particularly in healthy individuals, accounting for up to 46% of all biomedical imaging procedures [30]. Although the radiation doses associated with individual dentomaxillofacial examinations are relatively low compared to those used in the medical field, it is nevertheless essential to optimize this exposure in accordance with the “ALADAIP” principle [12]. This is because even small doses of X-ray exposure can increase the likelihood of adverse health outcomes [31]. This study’s findings suggest that low-dose protocols are feasible for diagnostic purposes in both imaging modalities, with PCD-CT offering superior image quality compared to CBCT [21]. These findings align with prior research supporting low-dose CBCT in dental implant imaging [13] and further highlight PCD-CT’s potential to reduce radiation exposure in clinical practice [22].

Our results indicate that PCD-CT provides superior image quality and reduced susceptibility to technical artifacts compared to CBCT. This advantage was observed consistently across both standard and low-dose protocols, with robust inter-reader agreement across all protocols, ranging from moderate to good. These findings are consistent with those of previous studies, which reported significantly higher image quality and approximately 30% improved contrast-to-noise ratios for PCD-CT compared to CBCT [22]. Moreover, a qualitative assessment of dentomaxillofacial structures based on Likert rating scales showed that, in a low-dose inter-modality comparison, PCD-CT reconstructions achieved superior depiction with higher ratings and inter-reader agreement (ICC > 0.6; *p* < 0.05) [22]. These results highlight the enhanced diagnostic clarity provided by PCD-CT while maintaining diagnostic accuracy and minimizing radiation exposure, which is further supported by the findings of this study’s low-dose intermodality comparison.

In this study, all PCD-CT scans were acquired in the ultra-high resolution mode with dose settings matching the CBCT scans to ensure comparability. The findings of this study indicate that PCD-CT scans reconstructed with a slice thickness of 0.2 mm, when using alongside established protocols, are a reliable and versatile imaging option for perioperative dental implant surgery. These approaches enabled detailed visualization of soft- and hard-tissue structures and reduced implant-related artifacts. In addition to overcoming several inherent limitations of CBCT, this technique could also reveal pathologies that would otherwise be obscured by metallic artifacts. This enhanced clarity may facilitate the early detection and diagnosis of these conditions, thus, potentially improving clinical outcomes especially in anatomically complex regions. Overall, this study highlights the clinical value of ultra-high resolution PCD-CT scans, offering a balanced combination of diagnostic interpretability and artifact reduction at low radiation dose.

To the best of our knowledge, this is the first study to evaluate the quantitative assessment of bone height and width in PCD-CT, specifically in the context of dental implant placement. The findings of this study demonstrate that PCD-CT provides accurate and reliable measurements, with minimal differences compared to CBCT. Moreover, PCD-CT fulfills the clinical precision standards necessary for implant planning, regardless of the radiation dose applied or the reader’s experience and subspecialty. This allows for a precise radiological assessment of osseous parameters essential for the long-term stability and successful integration of the implant to be performed prior to surgical intervention [32].

While this study design provides valuable insights, it is important to acknowledge the methodology’s inherent limitations. First, although pig mandibles are commonly used in dentomaxillofacial research, they do not fully replicate the anatomical conditions observed in human clinical settings. However, this limitation applies equally to both imaging modalities, ensuring the comparability of the acquired data. Second, the small sample size and the exclusive use of titanium as an implant material limit the generalizability of our findings. Further research with larger sample sizes is mandatory to investigate whether the observed differences between imaging modalities are applicable to a broader range of implant manufacturers, designs, materials, placement techniques, or anatomical variations in different regions of the mandible. Third, the higher cost, limited availability of PCD-CT scanners, and increased staffing and logistical requirements currently pose challenges to their implementation in dental practices. Moreover, validation in human subjects is essential to assess the clinical applicability of PCD-CT in dentomaxillofacial workflows, particularly in comparison with CBCT or other established radiological methods.

## Conclusions

This comparative ex vivo study demonstrates the potential of PCD-CT to impact dental implant imaging significantly. At equivalent radiation doses, PCD-CT offers several advantages over conventional CBCT, including excellent spatial resolution and a significant reduction in implant-related artifacts at all radiation exposure levels. Moreover, PCD-CT demonstrates comparable reliability in quantitative measurements essential for implant planning. With ongoing advancements in cost-effectiveness, accessibility, and validation in clinical trials, PCD-CT has the potential to emerge as a promising perioperative diagnostic modality. From a clinical standpoint, low-dose PCD-CT protocols, maintaining both quantitative and qualitative diagnostic accuracy in treatment planning, can play a significant role in enhancing patient safety by reducing radiation exposure. This study further highlights a more personalized imaging approach that balances radiation dose and diagnostic performance according to the indication-specific requirements.

## Data Availability

The datasets generated and analyzed during the current study are available from the corresponding author upon reasonable request.
